# Supplementary material for: Emergence of universality in the transmission dynamics of COVID-19
Source: Sci Rep. 2021 Sep 23;11:18891. doi: 10.1038/s41598-021-98302-3 (PMC8460722; doi:10.1038/s41598-021-98302-3)
Supplement: Supplementary file 1 — Supplementary Information. [file 41598_2021_98302_MOESM1_ESM.pdf]

Supplementary Information: Emergence of universality in the transmission dynamics of COVID-19

Ayan Paul, Jayanta Kumar Bhattacharjee, Akshay Pal, and Sagar Chakraborty

Universality in physical systems

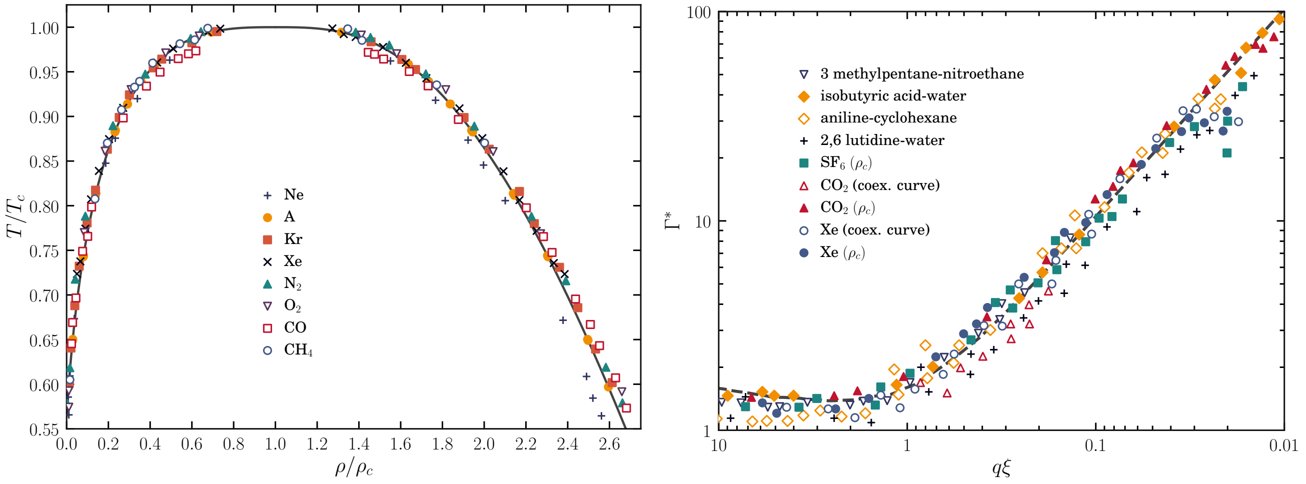

Figure 1. ***The emergence of universality in fluid systems****.The left panel is a reproduction of figure 2 of Ref. [1] which shows data collapse in the coexistence of liquid and vapour states for several fluids. The right panel is a reproduction of figure 10 of Ref. [2] and shows dynamical scaling for several fluids.*

In this section we discuss some examples of data collapse seen in physical system. The emergence of universality accompanied by a data collapse is quite common and has been observed for more than half a century. We begin our exposition with data collapse in the study of critical phenomenon [3–5] in fluids. We intend to keep the discussion only at a representative level to give some introduction to universality and data collapse and not make it an exhaustive survey which is not within the scope of this paper.

It is well known that if a gas is subjected to isothermal compression, then at a certain pressure it undergoes a phase transition to the liquid state while the pressure remains constant. Upon completion of the phase transition, the pressure increases very sharply on further compression while the volume increases too. If the temperature is raised and the process repeated, the same phenomenon occurs except that the parameter space over which the pressure and volume simultaneously rise grows smaller and smaller as the temperature is raised. At a particular temperature, $T = T_c$, this parameter space reduces to a point and at higher temperatures the liquefaction does not occur however high the pressure is. The temperature, T_c, is the critical temperature and in its vicinity the fluid shows a large amount of fluctuation as it lies between a state of reasonable order, the liquid state, and a state of disorder, which is the gaseous state.

The fluctuations in density very close to the critical point actually mean that some short-lived liquid state bubbles are produced in the gas. As the critical point is reached these fluctuations become long lived and infinitely long ranged as one has a liquid-vapour coexistence. The infinite lifetime of fluctuations and the infinite range of correlation mean that this phenomenon cannot be sensitive to which gas is being considered. Whether it be Carbon Dioxide, Oxygen, Nitrogen, Xenon or Sulphur Hexafluoride, they will all behave in the same way. This is known as universality and, hence, means that if we draw the equation of state of any gas near the critical point with pressure scaled by the critical pressure and the volume scaled by the critical volume, then we will get just one curve. The critical volumes, critical temperatures and critical pressures are all different for the different materials but the scaled equation of state is the same as can be seen from Figure 1. If P, V and T are the pressure,

volume and temperature of a fluid with a and b being constants specific to the fluid, then

$$\left(p + \frac{a}{V^2}\right)(V - b) = RT \longrightarrow \left(\pi + \frac{3}{\phi^2}\right)(3\phi - 1) = 8\tau, \tag{1}$$

where $\pi = P/P_c$, $\phi = V/V_c$ and $\tau = T/T_c$ with P_c, V_c and T_c being the pressure volume and temperature at the critical point. The equation of state thus collapses to a single equation independent of the nature of the fluid.

An identical situation holds for the dynamics which probes the lifetime of the fluctuations. The experimental technique for probing fluctuations directly is frequency resolved light scattering. The intensity I as a function of frequency, ω, is what is measured and at a given temperature and has a Lorentzian shape. As the temperature is lowered towards the critical point, both the peak (at $\omega = 0$) and the width at half-maximum increase with decreasing temperature, both headed for infinitely large values at the critical point. Different materials show different Lorentz distributions at different temperatures. If we denote the intensity at a particular frequency and a particular temperature, T, by $I(\omega, \Delta T)$, with $\Delta T = T - T_c$, then for different materials and different sets of ΔT one has different $I(\omega, \Delta T)$ vs ω distributions. However, if one plots $I(\omega, \Delta T)/I(0, \Delta T)$ vs. ω/ω_0 where ω_0 is the characteristic frequency for a given material, then the data for hundreds of individual distributions collapse on a single distribution. This phenomenon is called dynamic scaling [3–5] and is displayed in Figure 1.

This phenomenon of universal data collapse is ubiquitous: It appears in various forms in network dynamics [6], neuronal network [7], brain [8,9], supercritical fluids [10], amorphous solids [11], Bose–Einstein condensate [12], jamming transition [13], granular media [14, 15], financial markets [16], metallic liquids [17], etc. Thus, in some sense, it is not surprising that this intriguing phenomenon be present in the transmission of infectious diseases as well.

Comparisons between different models

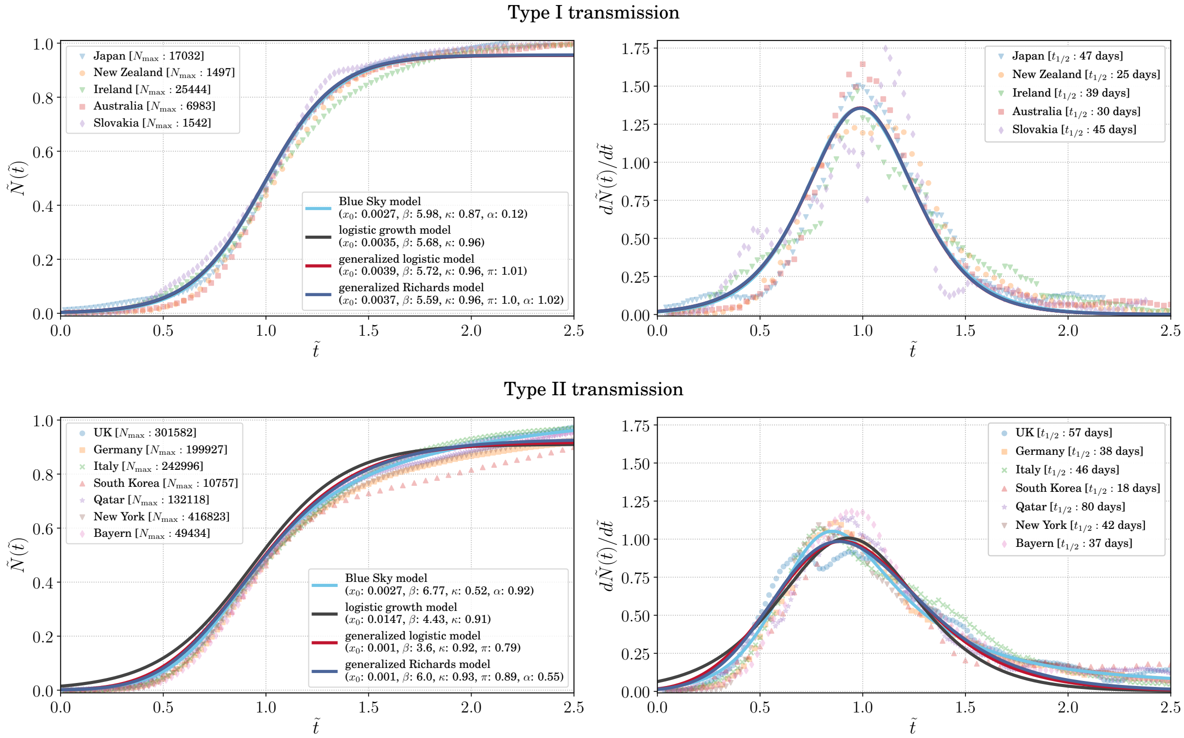

Figure 2. ***A comparison of the variants of the logistic model.*** *The panels on the left show $\tilde{N}(\tilde{t})$ vs. $\tilde{t}$ distribution for Type I and Type II transmission dynamics; and the right panels shows the $d\tilde{N}(\tilde{t})/d\tilde{t}$ vs. $\tilde{t}$ plot for the same. We fit four models to the data – the logistic growth model, the generalized logistic model, the generalized Richards model and the Blue Sky model. While the fit to data for all the models are the same for Type I transmission where all the models go to the limit of being a logistic growth model, for Type II transmission the Blue Sky model can explain the data better than the other models.*

In this section we compare some models that have been used commonly in epidemiology to quantify transmission dynamics of diseases including COVID-19 [18]. All the models that we describe here are variants of the logistic growth model. The

logistic growth model (LGM) is defined as:

$$\frac{dN(t)}{dt} = \beta N(t)\left(1 - \frac{N(t)}{\kappa}\right). \tag{2}$$

We define $\tilde{t} = t/t_{1/2}$ and $\tilde{N}(t) = N(t)/N_{\max}$, and by setting $\tilde{\beta} = \beta t_{1/2}$ and $\tilde{\kappa} = \kappa/N_{\max}$, we get the rescaled equation:

$$\frac{d\tilde{N}(\tilde{t})}{d\tilde{t}} = \tilde{\beta}\tilde{N}(\tilde{t})\left(1 - \frac{\tilde{N}(\tilde{t})}{\tilde{\kappa}}\right). \tag{3}$$

We see that β scales as the time dimension and κ scales as the number of cases. This makes the dimensional quantities, $\tilde{\beta}$ and $\tilde{\kappa}$, independent of any characteristic timescale or size of the system. $\tilde{\kappa} = 1$ in the LGM.

The generalized logistic model (GLM) is given by:

$$\frac{dN(t)}{dt} = \beta N(t)^{\pi}\left(1 - \frac{N(t)}{\kappa}\right). \tag{4}$$

Similar rescaling as above leads to the rescaled parameters $\tilde{\beta} = \beta t_{1/2} N_{\max}^{\pi-1}$ and $\tilde{\kappa} = \kappa/N_{\max}$ leading to the rescaled GLM being:

$$\frac{d\tilde{N}(\tilde{t})}{d\tilde{t}} = \tilde{\beta}\tilde{N}(\tilde{t})^{\pi}\left(1 - \frac{\tilde{N}(\tilde{t})}{\tilde{\kappa}}\right). \tag{5}$$

The generalized Richards model (GRM) is given by:

$$\frac{dN(t)}{dt} = \beta N(t)^{\pi}\left[1 - \left(\frac{N(t)}{\kappa}\right)^{\alpha}\right]. \tag{6}$$

For $\pi = 1$, it reduces to the standard Richards model [19]. We see that the scaling is similar to the GLM with $\tilde{\beta} = \beta t_{1/2} N_{\max}^{\pi-1}$ and $\tilde{\kappa} = \kappa/N_{\max}$; the rescaled GRM is:

$$\frac{d\tilde{N}(\tilde{t})}{d\tilde{t}} = \tilde{\beta}\tilde{N}(\tilde{t})^{\pi}\left[1 - \left(\frac{\tilde{N}(\tilde{t})}{\tilde{\kappa}}\right)^{\alpha}\right]. \tag{7}$$

In Figure 2 we show a comparison between all the models we list here and the Blue Sky model (BSM). The two upper panels are for transmission of Type I. The LGM fits this data quite well and all the other models acquire the parameter values that take them to the limit of being an LGM. The two lower panels are for transmission of Type II. Here we clearly see that the BSM fits the data much better that the other models. Especially for the tail of distribution past the peak of the $d\tilde{N}(\tilde{t})/d\tilde{t}$ vs. $\tilde{t}$ plot where $\tilde{t} > \tilde{t}_{\text{peak}}$, the BSM model allows for larger $d\tilde{N}(\tilde{t})/d\tilde{t}$ compared to the other models hence being able to replicate transmission of Type II where the spread of the disease lingers on.

The deep neural network architechture

In our model-agnostic approach we used a deep neural network (DNN) [20,21] to fit the data. A DNN is a universal function generator. The reason we chose a DNN over other machine learning frameworks (i.e. decisions trees, random forests, SVM etc.) is the ease with which it can be use for a regression to noisy data to extract the underlying function. We will describe in brief the choice of the network architechture.

We started with a small DNN with 1 input node for $\tilde{t}$, 2 hidden layers, each with 4 nodes and 1 output layer for $\tilde{N}(\tilde{t})$. All layers were fully connected as shown in Figure 3. The activation function used was the *sigmoid* function. The solution to the LM is a *sigmoid* function and hence the transmission of Type 1 can be fairly explained with a just one sigmoid function that connects the input $\tilde{t}$ to the output $\tilde{N}(\tilde{t})$. This is not possible though for transmission of Type II which shows clear deviation from the LM. However, in the model agnostic approach we did not want to introduce any model biases through assumptions of functional form. Hence we used a larger (and deeper) neural network with 33 parameters to begin with. We scaled up the network to 3 hidden layers each with 16 nodes to check for the stability of our approach. The regression with the DNN with 2 hidden layers with 4 nodes each gave almost the same fit as the one with 3 hidden layers with 16 nodes each having a total of 593 parameters. To keep our method as general as possible and not introduce model biases from the activation function we decided to use the larger DNN with 3 hidden layers each having 16 nodes.

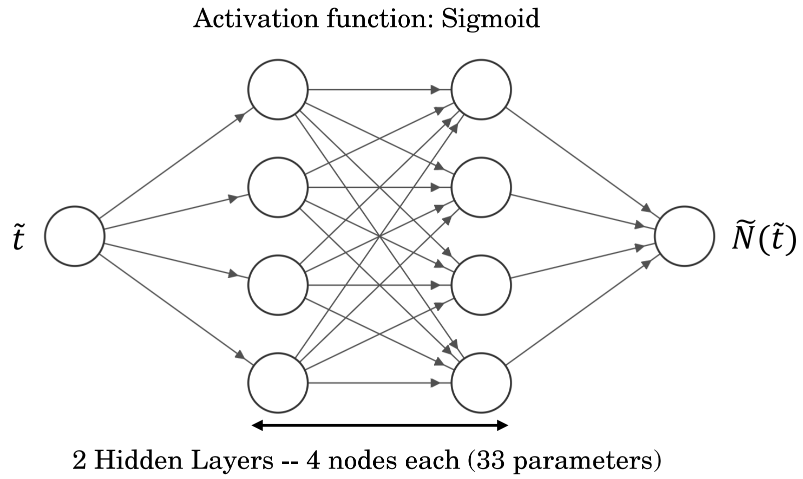

Figure 3. ***A representative architechture of the DNN used for the model-agnostic analysis to fit $\tilde{N}(\tilde{t})$ as a function of $\tilde{t}$.*** *For the final analysis we used a similar architechture with 3 layers each with 16 nodes to keep the analysis as model-agnostic as possible. To regulate the training of the DNN we used an early callback procedure which stops the training when the validation loss stops decreasing thus avoiding training biases.*

In addition, we tested the DNN with other activation function like *tanh* and *relu*. For *tanh* a somewhat wider and deeper network was needed to fit to the data. With the *relu* activation the network needed to be very large. Hence, we used the *sigmoid* activation function for the final DNN architecture.

Even though the DNN we used was not so large we used an early callback algorithm to stop the training of the network when the validation loss stopped decreasing to avoid overtraining and chose the epoch where the validation loss was the least. The loss function that we used was a simple mean square error function which measures the L_2 distance of the fit from the data. The training time was a few seconds with the early stopping coming into effect at about 100-150 epochs. The trained DNN was used for making prediction for the ongoing phases

References

1. Guggenheim, E. A. The Principle of Corresponding States. *The J. Chem. Phys.* **13**, 253–261, DOI: 10.1063/1.1724033 (1945).
2. Swinney, H. L. & Henry, D. L. Dynamics of Fluids near the Critical Point: Decay Rate of Order-Parameter Fluctuations. *Phys. Rev. A* **8**, 2586–2617, DOI: 10.1103/PhysRevA.8.2586 (1973).
3. Hohenberg, P. C. & Halperin, B. I. Theory of dynamic critical phenomena. *Rev. Mod. Phys.* **49**, 435–479, DOI: 10.1103/RevModPhys.49.435 (1977).
4. Stanley, H. E. Scaling, universality, and renormalization: Three pillars of modern critical phenomena. *Rev. Mod. Phys.* **71**, S358–S366, DOI: 10.1103/RevModPhys.71.S358 (1999).
5. LÜbeck, S. Universal scaling behavior of non-equilibrium phase transitions. *Int. J. Mod. Phys. B* **18**, 3977–4118, DOI: 10.1142/S0217979204027748 (2004).
6. Barzel, B. & Barabási, A.-L. Universality in network dynamics. *Nat. Phys.* **9**, 673–681, DOI: 10.1038/nphys2741 (2013).
7. Friedman, N. *et al.* Universal Critical Dynamics in High Resolution Neuronal Avalanche Data. *Phys. Rev. Lett.* **108**, 208102, DOI: 10.1103/PhysRevLett.108.208102 (2012).
8. Bhattacharya, J. & Petsche, H. Universality in the Brain While Listening to Music. *Proceedings: Biol. Sci.* **268**, 2423–2433 (2001). URL: http://www.jstor.org/stable/3067747.
9. Serafino, M. *et al.* True scale-free networks hidden by finite size effects. *Proc. Natl. Acad. Sci.* **118**, DOI: 10.1073/pnas.2013825118 (2021).
10. Ha, M. Y., Yoon, T. J., Tlusty, T., Jho, Y. & Lee, W. B. Universality, Scaling, and Collapse in Supercritical Fluids. *The J. Phys. Chem. Lett.* **11**, 451–455, DOI: 10.1021/acs.jpclett.9b03360 (2020).
11. Ulrich, S., Upadhyaya, N., Opheusden, B. v. & Vitelli, V. Shear shocks in fragile networks. *Proc. Natl. Acad. Sci.* **110**, 20929–20934, DOI: 10.1073/pnas.1314468110 (2013).

12. Klinder, J., Keßler, H., Wolke, M., Mathey, L. & Hemmerich, A. Dynamical phase transition in the open Dicke model. *Proc. Natl. Acad. Sci.* **112**, 3290–3295, DOI: 10.1073/pnas.1417132112 (2015).

13. Goodrich, C. P., Liu, A. J. & Sethna, J. P. Scaling ansatz for the jamming transition. *Proc. Natl. Acad. Sci.* **113**, 9745–9750, DOI: 10.1073/pnas.1601858113 (2016).

14. Manna, S. S. Two-state model of self-organized criticality. *J. Phys. A: Math. Gen.* **24**, L363–L369, DOI: 10.1088/0305-4470/24/7/009 (1991).

15. Denisov, D. V., Lörincz, K. A., Uhl, J. T., Dahmen, K. A. & Schall, P. Universality of slip avalanches in flowing granular matter. *Nat. Commun.* **7**, 10641, DOI: 10.1038/ncomms10641 (2016).

16. Denys, M., Gubiec, T., Kutner, R., Jagielski, M. & Stanley, H. E. Universality of market superstatistics. *Phys. Rev. E* **94**, 042305, DOI: 10.1103/PhysRevE.94.042305 (2016).

17. Blodgett, M. E., Egami, T., Nussinov, Z. & Kelton, K. F. Proposal for universality in the viscosity of metallic liquids. *Sci. Reports* **5**, 13837, DOI: 10.1038/srep13837 (2015).

18. Wu, K., Darcet, D., Wang, Q. & Sornette, D. Generalized logistic growth modeling of the COVID-19 outbreak: comparing the dynamics in the 29 provinces in China and in the rest of the world. *Nonlinear Dyn.* **101**, 1561–1581, DOI: 10.1007/s11071-020-05862-6 (2020).

19. Richards, F. J. A Flexible Growth Function for Empirical Use. *J. Exp. Bot.* **10**, 290–301, DOI: 10.1093/jxb/10.2.290 (1959).

20. Bengio, Y., Courville, A. & Vincent, P. Representation Learning: A Review and New Perspectives. *IEEE Transactions on Pattern Analysis Mach. Intell.* **35**, 1798–1828, DOI: 10.1109/TPAMI.2013.50 (2013).

21. Schmidhuber, J. Deep learning in neural networks: An overview. *Neural Networks* **61**, 85 – 117, DOI: 10.1016/j.neunet.2014.09.003 (2015).
